# Molecular Genetic Characteristics of Plasmid-Borne *mcr-9* in *Salmonella enterica* Serotype Typhimurium and Thompson in Zhejiang, China

**DOI:** 10.3389/fmicb.2022.852434

**Published:** 2022-03-17

**Authors:** Jianzhong Fan, Heng Cai, Youhong Fang, Jintao He, Linghong Zhang, Qingye Xu, Yunxing Yang, Sebastian Leptihn, Yunsong Yu, Dongdong Zhao, Xiaoting Hua

**Affiliations:** ^1^Department of Laboratory Medicine, Affiliated Hangzhou First People’s Hospital, Zhejiang University School of Medicine, Hangzhou, China; ^2^Department of Infectious Diseases, Sir Run Run Shaw Hospital, Zhejiang University School of Medicine, Hangzhou, China; ^3^Key Laboratory of Microbial Technology and Bioinformatics of Zhejiang Province, Hangzhou, China; ^4^Regional Medical Center for National Institute of Respiratory Diseases, Sir Run Run Shaw Hospital, School of Medicine, Zhejiang University, Hangzhou, China; ^5^Department of Gastroenterology, The Children’s Hospital, Zhejiang University School of Medicine, National Clinical Research Center for Child Health, Hangzhou, China; ^6^Zhejiang University-University of Edinburgh (ZJU-UoE) Institute, Zhejiang University, Haining, China

**Keywords:** *mcr-9*, colistin, IncHI2 plasmid, *Salmonella* Typhimurium, *Salmonella* Thompson

## Abstract

*Salmonella enterica* is a zoonotic food-borne pathogen threatening public health around the world. As is the case with many other pathogens, the spread of mobilized colistin resistance (*mcr*) alleles is of grave concern. In this study, totally 689 clinical *Salmonella* isolates were collected from a local hospital in Hangzhou, Zhejiang Province, China between 2009 and 2018. Resistance genes were screen by PCR. Two *mcr-9-*positive *Salmonella* strains S15 and S639 were identified which belong to serotype Typhimurium and Thompson, respectively. We observed that both *mcr-9* genes were located on conjugative IncHI2 plasmids which encoded numerous resistance genes, likely facilitating the dissemination of *mcr-9* by co-resistance mechanisms. The *mcr-9* cassettes encoded on the two plasmids were not identical: downstream of the *mcr-9* genes, we found IS*1* on one plasmid (pS15), while the other had a *WbuC*-IS*26* (pS639). Despite the presence of *mcr-9* cassettes, the strains were not rendered colistin resistant. Yet, it is of epidemiological importance to implement surveillance to be able to observe and possibly control the spread of *mcr-9* due to its potential to mediate resistance to the last-resort antibiotic colistin.

## Introduction

Colistin is an effective antibiotic for the treatment of infections caused by multidrug-resistant Gram-negative bacteria as one of the last-resort therapeutic options (Nation and Li, 2009). Since the plasmid-encoding colistin-mediated resistance gene *mcr-1* was reported in *Escherichia coli* of animal origin in China (Liu et al., 2016), plasmid-borne *mcr* alleles have gained increasing attention and have been extensively researched. Successively, *mcr-2* to *mcr-10* have been identified, most from animals (Carroll et al., 2019; Lima et al., 2019; Wang et al., 2020). According to the current data, *mcr-1* and *mcr-9* are the most common colistin resistance cassettes with *mcr-9* prevalent in *Salmonella enterica* (Ling et al., 2020). *Salmonella enterica* is an important zoonotic pathogen, which can disseminate between animals and people through contaminated food (Lima et al., 2019). Nontyphoidal *Salmonella* usually causes self-limited enterocolitis with diarrhea. Occasionally an infection with the pathogen can result in more severe diseases including bloodstream infections especially in young children, the elderly, and immunocompromised people (Crump et al., 2015). Thus, the increasing antimicrobial resistance in *Salmonella* species needs to be monitored (Lozano-Leon et al., 2019).

In a previous study, we focused on the prevalence of the *mcr-1* gene in 689 clinical *Salmonella* isolates in a local hospital and six *mcr-1* positive strains were identified (Fan et al., 2020). Five strains belonged to *S.* Typhimurium and one belonged to *S.* Indiana. In this work, we have screened the *Salmonella* isolates for other *mcr* alleles (*mcr-*2 to *mcr-*10). Here, we identified two plasmid-borne *mcr-9* in *Salmonella* Typhimurium and *Salmonella* Thompson. To our knowledge, this is the first detailed description of *mcr-9* plasmid of *Salmonella* Thompson. In this work, we characterized the composition of the *mcr-9* carrying plasmids and the genetic environment surrounding the *mcr-9* cassettes, which differed in the two plasmids.

## Materials and Methods

### Clinical Isolates and Identification

*Salmonella* clinical isolates were isolated from patients’ specimens such as blood, feces, synovial fluid and pus from abdominal and skin and soft tissue infections in the First People’s Hospital of Hangzhou, Zhejiang Province, China, between 2009 and 2018. Bacterial species were identified by the automated Vitek 2 system (BioMérieux, Marcy-l’Étoile, France) and matrix-assisted laser desorption ionization time of flight mass spectrometry (MALDI-TOF MS) (Bruker, Bremen, Germany). *Salmonella* serotyping was identified by slide agglutination with specific antisera (Tianrun Bio-Pharmaceutical Co., Ltd., Ningbo, China) according to the White-Kauffmann-Le Minor scheme (9th edition).

### *mcr* Alleles Screened by PCR and Sequencing

All *Salmonella* isolates were screened for *mcr-2* to *mcr-10* by using PCR with corresponding pairs of primers (Table 1). The amplification products were subsequently sequenced by Sanger sequencing for confirmation.

**Table 1:** The primers used in this study.

Primer	Sequence of primer (from 5' to 3')	Usage
mcr-2-F	CAAGTGTGTTGGTCGCAGTT	Screening for *mcr* alleles
mcr-2-R	TCTAGCCCGACAAGCATACC	
mcr-3-F	TTGGCACTGTATTTTGCATTT	
mcr-3-R	TTAACGAAATTGGCTGGAACA	
mcr-4-F	ATTGGGATAGTCGCCTTTTT	
mcr-4-R	TTACAGCCAGAATCATTATCA	
mcr-5-F	ATGCGGTTGTCTGCATTTATC	
mcr-5-R	TCATTGTGGTTGTCCTTTTCTG	
mcr-6-F	AGCTATGTCAATCCCGTGAT	
mcr-6-R	ATTGGCTAGGTTGTCAATC	
mcr-7-F	GCCCTTCTTTTCGTTGTT	
mcr-7-R	GGTTGGTCTCTTTCTCGT	
mcr-8-F	TCAACAATTCTACAAAGCGTG	
mcr-8-R	AATGCTGCGCGAATGAAG	
mcr-9-F	TTCCCTTTGTTCTGGTTG	
mcr-9-R	GCAGGTAATAAGTCGGTC	
mcr-10-F	GGACCGACCTATTACCAGCG	
mcr-10-R	GGCATTATGCTGCAGACACG	
XH104-F	AAAGTCATCATCCCCTAATGCTTTTG	Verification of transconjugants
XH104-R	TGACAGTATTAGGATTTGCGGTTG	
S15-mcr9.1-F	TGTATGAATCCCGCCTGAAGGGA	
S15-mcr9.1-R	TGCAGCGAATAAGGCAATCATAA	

### Antimicrobial Susceptibility Testing

Antimicrobial susceptibility testing was performed by broth microdilution, including colistin, ampicillin, amoxicillin, piperacillin-tazobactam, cefazolin, cefoxitin, ceftriaxone, cefepime, ceftazidime, aztreonam, ertapenem, imipenem, meropenem, amikacin, gentamicin, kanamycin, ciprofloxacin, levofloxacin, tigecycline, tetracycline, trimethoprim-sulfamethoxazole, and fosfomycin. The minimum inhibitory concentration (MIC) of nitrofurantoin was performed using E-test method. Antimicrobial susceptibility testing of the transconjugants was performed by broth microdilution, including colistin, ampicillin, amoxicillin, piperacillin-tazobactam, amikacin, gentamicin, kanamycin, and tetracycline.

The results of antimicrobial susceptibility testing were interpreted by Clinical and Laboratory Standards Institute guidelines (CLSI) (M100, 30th ed.; CLSI, 2020), except that colistin and tigecycline were used the European Committee on Antimicrobial Susceptibility Testing (EUCAST) breakpoints v8.1. The quality control strain was *E. coli* ATCC 25922.

### Genome Sequencing and Analysis

Genomic DNA of two *mcr-9*-positive strains S15 and S639 were sequenced by HiSeq (Illumina, San Diego, CA, United States) and MinION sequencer (Oxford Nanopore Technologies, Oxford, United Kingdom). The short read and long read sequence data were hybrid *de novo* assembled by Unicycler v0.4.8 (Wick et al., 2017). The gene sequences were annotated by Prokka (Seemann, 2014) and NCBI Blast (Camacho et al., 2009). Resistance genes and insertion sequence (IS) were identified by BacAnt (Hua et al., 2021). Multi-locus sequence typing (MLST) was identified by using mlst.^1^ The gene sequences were compared and visualized by Easyfig 2.2.5 (Sullivan et al., 2011) and BRIG-0.95 (Alikhan et al., 2011).

### Conjugation Experiments

Conjugation assays were conducted by using rifampicin-resistant *Salmonella* strain XH1984 and *E. coli* strain EC600 as the recipient strain and strain S15 as the donor. The Mueller-Hinton agar plates containing rifampicin (100 μg/ml) and ampicillin (4 μg/ml for S15 and XH1984; 32 μg/ml for S15 and EC600) were used for selection. The successful transconjugants of S15 and XH1984 were verified by PCR using two pairs of primers: XH104-F and XH104-R; S15-mcr9.1-F and S15-mcr9.1-R (Table 1). The former pair of primers was used to identify XH1984 and the latter was used to identify pS15. The transconjugants of S15 and EC600 were verified by S15-mcr9.1 primers and MALDI-TOF MS. The conjugation frequency of pS15 was determined.

### Phylogenetic Trees of *mcr-9*-Carrying *Salmonella*

The assembled *mcr-9* carrying *S.* Thompson and *S.* Typhimurium genomes were downloaded from NCBI and annotated using prokka 1.13 (Seemann, 2014). The maximum likelihood phylogenetic tree was constructed with IQTree 2.1.2 (Nguyen et al., 2015) from a multiple alignment of the core genomes generated by Roary 3.7.0 (Page et al., 2015). The trees were visualized with ggtree (Yu, 2020) and ggtreeExtra (Xu et al., 2021) in R.

## Results

### Screening for *mcr-2* to *mcr-10*

We previously screened 689 clinical *Salmonella* isolates from hospital patient specimens for the presence of the colistin resistance gene *mcr-1* (Fan et al., 2020). This follow-up study, we have screened all isolates for other types of *mcr* genes, including *mcr-2* to *mcr-10*. While none of the strains contained any *mcr-2* to *mcr-8* or *mcr-10* genes, we found two (0.29%) *mcr-9*-positive *Salmonella* spp. strains, S15 and S639. The strain S15 was isolated from the stool of a 53-year-old female patient in 2011, while S639 was a stool sample isolate from a 24-year-old woman obtained in 2018. Both patients came to the outpatient service with symptoms of diarrhea.

### Results of Antimicrobial Susceptibility Testing

The antimicrobial susceptibility results are displayed in Table 2. Two *mcr-9*-positive strains were both resistant to ampicillin, amoxicillin, and tetracycline. S639 was additionally resistant to cefazolin, cefoxitin, ceftriaxone, ceftazidime, aztreonam, amikacin, kanamycin, ciprofloxacin, levofloxacin, and trimethoprim-sulfamethoxazole. Both strains were sensitive to colistin, cefepime, ertapenem, imipenem, meropenem, gentamicin, tigecycline, and nitrofurantoin.

**Table 2:** Summary of antimicrobial susceptibility testing.

Antibiotics (μg/ml)	Strains					
	S15	S639	XH1984	XH1984-pS15	EC600	EC600-pS15
Colistin	0.5	0.5	1	1	0.06	0.06
Ampicillin	512	>2,048	1	>64	8	>128
Amoxicillin	32	64	1	>64	16	>128
Piperacillin-tazobactam	4/4	32/4	2/4	4/4	4/4	8/4
Cefazolin	2	>1,024				
Cefoxitin	4	128				
Ceftriaxone	0.5	8				
Cefepime	0.25	0.5				
Ceftazidime	0.5	>64				
Aztreonam	0.06	32				
Ertapenem	0.032	0.25				
Imipenem	0.5	1				
Meropenem	0.06	0.125				
Amikacin	4	32	2	2	4	4
Gentamicin	1	0.5	0.25	0.25	0.25	0.25
Kanamycin	2	32	2	2	16	16
Ciprofloxacin	0.032	0.5				
Levofloxacin	0.125	1				
Tigecycline	0.25	0.5				
Tetracycline	32	256	1	32	4	>32
Nitrofurantoin	24	16				
Trimethoprim-sulfamethoxazole	2/38	>32/608				
Fosfomycin	4	128				

### Whole-Genome Sequencing Analysis

The serotype of S15 was *Salmonella* Typhimurium (O4:Hi), belonging to ST 34. The strain contained a single plasmid only which we called pS15. The plasmid encoded the *mcr-9* gene which showed a 100% identity and coverage to the previously reported *mcr-9* in *Enterobacterales* (WP_044704969.1). The genome size of IncHI2 plasmid pS15 was 266,098 bp and the GC content was 46%. This strain contains 12 resistance genes on the plasmid and 20 resistance genes on the chromosome (Table 3).

**Table 3:** Resistance genes in two strains.

Strains		ST	Plasmid type	Resistance genes in plasmid	Resistance genes in chromosome
S15	ST34	IncHI2, IncHI2A	*terW,terZ,merD,merB,merR_Ps,* *bla*_TEM-1_ *,tet(A),dfrA16,aadA2,* *mcr-9,pcoS,pcoE*	*sinH,golS,golT,mdsB,pcoE,* *pcoS,pcoD,pcoC,pcoB,pcoA,silP,silB,silF,silC,silS,silE,arsC_gluta,arsB_pKW301,arsA,arsR_pKW301*	S639	ST26	IncHI2, IncHI2A	*terW,terZ,aadA2,sul1,bla*_TEM-1_, *merT,merA,merE,mph(A),sul1,* *bla*_OXA-10_*,aacA34,arr-3,aph(6)-ld,aph(3″)-lb,sul2,catA2,tet(D),* *pcoE,pcoS,mcr-9.1,aph(6)-ld,* *aph(3″)-lb,dfrA19,sul1,bla*_DHA-1_, *qnrB4*	*sinH,golS,golT,mdsB*

The serotype of S639 was *Salmonella* Thompson (O7:Hk:H1,5), belonging to ST 26. Again, this strain contained only a single plasmid which we called pS639. Here, the *mcr-9* exhibited 100% identity and coverage to another colistin resistance gene in the *Enterobacterales*, WP_001572373, *mcr-9.1*. This sequence was missing a single codon (for tryptophan) right before the STOP codon compared to *mcr-9* in S15. The genome size of IncHI2 plasmid pS639 was 308,491 bp and the GC content was 48%. This strain contains 27 resistance genes on the plasmid while only four resistance genes are found on the chromosome (Table 3).

Most of the chromosome-encoded resistance genes in S15 and S639 are related to metal resistance aside from the multidrug efflux RND transporter gene *mdsB*. The resistance genes in two plasmids differ greatly from each other: for example, pS15 contains genes associated with resistance to tellurium (*terW* and *terZ*), mercury (*mer* gene cluster), β-lactams (*bla*_TEM-1_), tetracycline [*tet(A)*], trimethoprim (*dfrA16*), streptomycin (*aadA2*), colistin (*mcr-9*), and copper (*pcoS* and *pcoE*). Plasmid pS639 encodes genes mediating resistance to all substances which are facilitating resistance in pS15, some of which were different alleles like *dfrA19* and *tet(D).* Additionally, pS639 encodes genes associated with resistance to sulfonamide (*sul1* and *sul2*), macrolide [*mph(A)*], β-lactams (*bla*_OXA-10_), aminoglycoside (*aacA34*), rifamycin (*arr-3*), streptomycin [*aph(6)-ld* and *aph(3″)-lb*], chloramphenicol (*catA2*), cephalosporin (*bla*_DHA-1_), and quinolone (*qnrB4*). This abundance of additional ARGs might explain why S639 exhibited higher MIC values and a wider resistance to more antibiotics than S15.

### Comparison of the Plasmid Sequences

**Figure 1:**
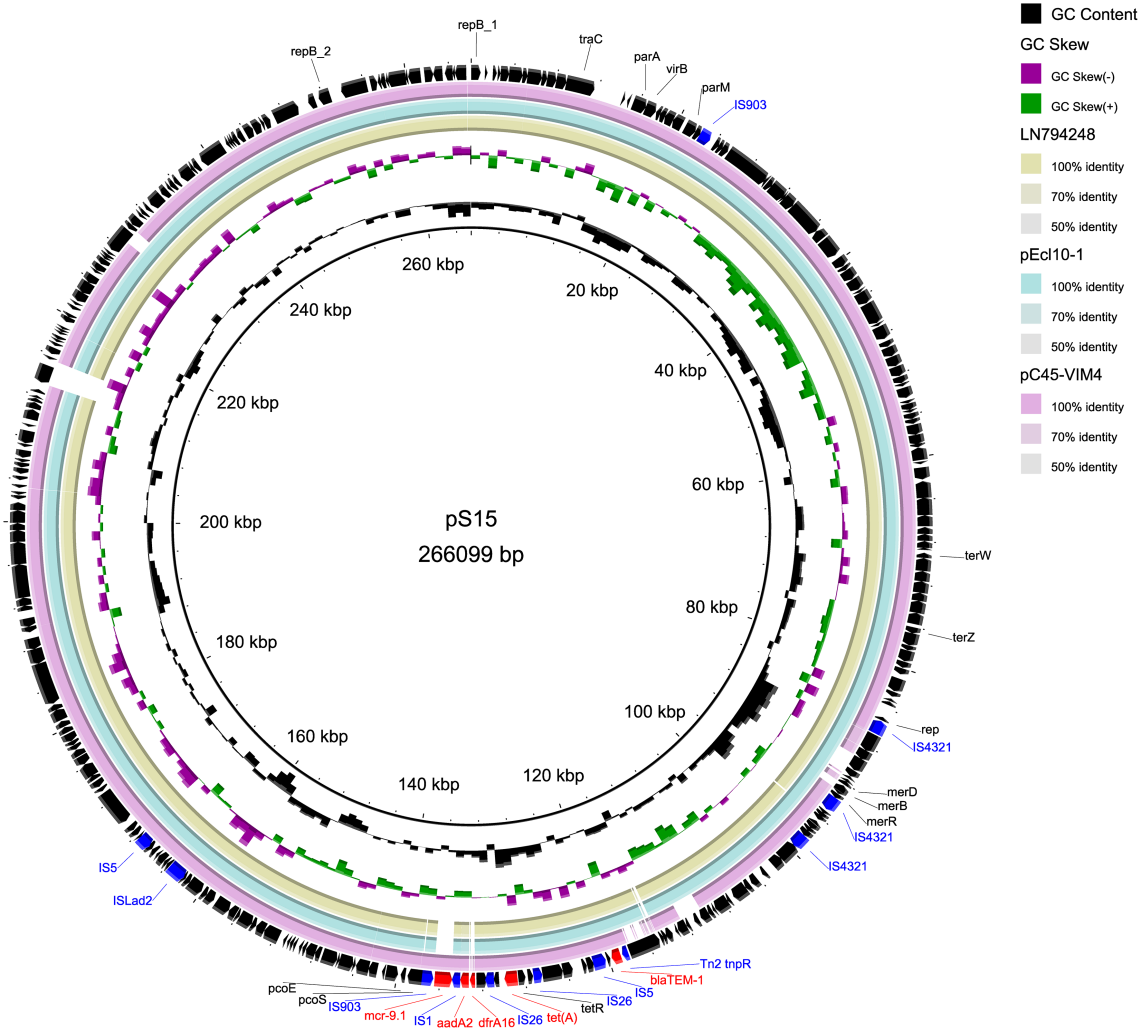
Comparison of circular structures of pS15 and its similar plasmids. Arrows indicate the direction of predicted transcription of each gene. Red arrows indicate antibiotic resistance genes. Blue arrows indicate insertion sequences.

**Figure 2:**
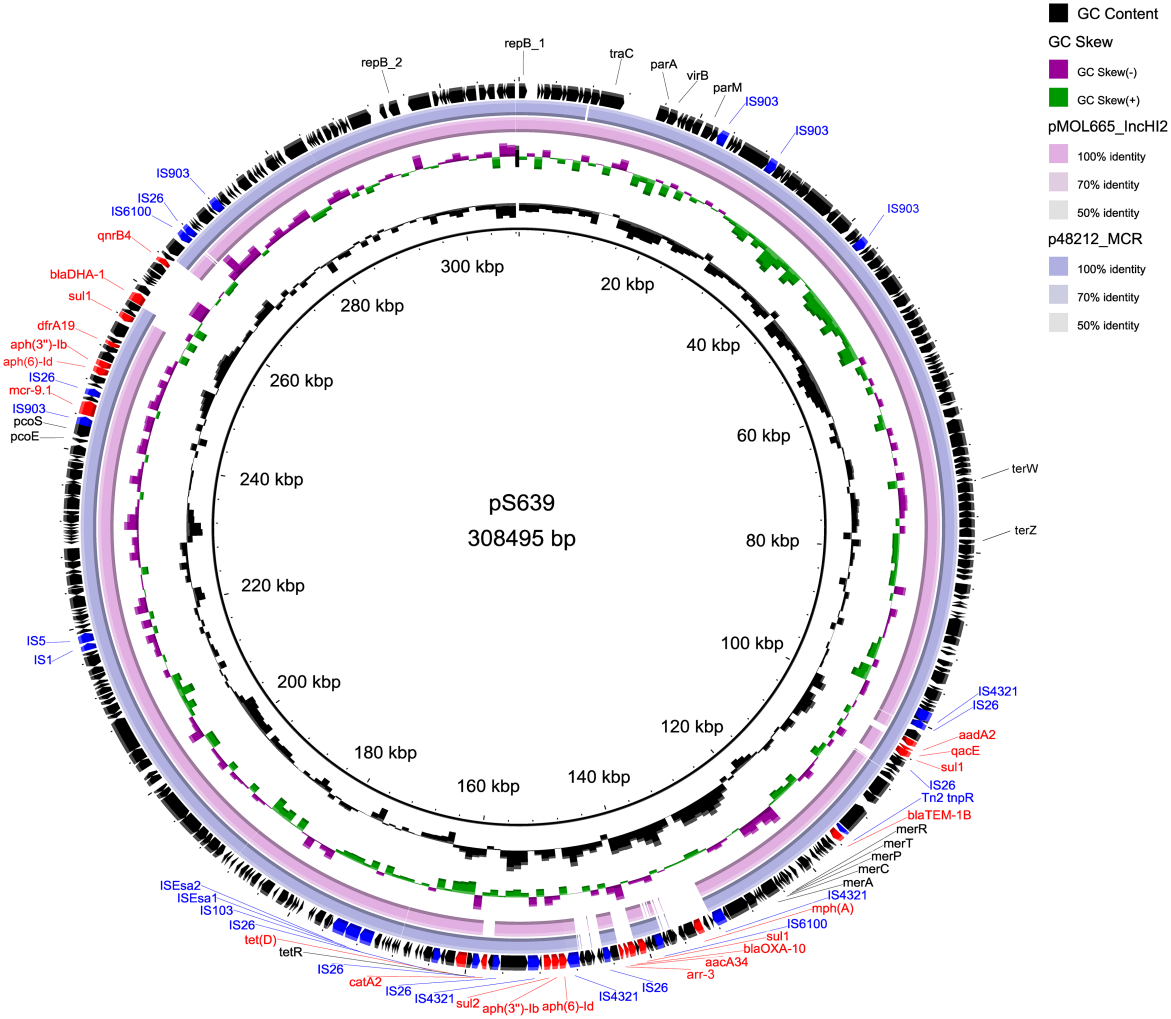
Comparison of circular structures of pS639 and its similar plasmids. Arrows indicate the direction of predicted transcription of each gene. Red arrows indicate antibiotic resistance genes. Blue arrows indicate insertion sequences.

The two plasmids we found are similar to other IncHI2 plasmids. Table 4 lists plasmids for comparison, some of which share high query coverage and identity with pS15 and pS639 from different species. The backbone structures of pS15, pEcl10-1 (CP048704), sLN794248 (LN794248), and pC45-VIM4 (LT991958) are closely related (Figure 1). The main differences between them are the resistance gene cluster regions where most insertion elements (ISs) were located. Although pEcl10-1 and sLN794248 were more similar to pS15 in their sequence, they do not contain *mcr-9.1* and *dfrA16*. Plasmid pC45-VIM4 encodes a *mcr-9.1* gene but shows more differences in the 90–140 kbp region of pS15 compared to other plasmids. When comparing pS15 to three other plasmids, pS15 contains a gene encoding group II intron reverse transcriptase/maturase (around 210 kb). Two resistance gene clusters are present in pS639, found in two sections, from ~100 to 180 kb and from ~240 to 270 kb, respectively (Figure 2). The first section shares some similarities but also substantial differences with two other plasmid sequences, p48212_MCR (CP059413) and pMOL665_IncHI2 (OU015720). pS639 additionally encodes *mph(A)*, *bla*_OXA-10_, *aacA34*, *aar-3,* and *catA2*, genes which are able to facilitate the resistance to macrolide, β-lactam, aminoglycoside, rifamycin, and chloramphenicol. In the other resistance gene cluster which includes *mcr-9.1*, the main difference is an insertion of the two resistance genes *bla*_DHA-1_ and *qnrB4* while a gene cluster encoding phage shock protein is also present.

**Table 4:** Comparison of two *mcr-9-*positive plasmids with similar plasmids.

Plasmid	Similar plasmid	Species	Query coverage (%)	Identity (%)
pS15 (*Salmonella* Typhimurium)	pEcl10-1 (CP048704)	*Enterobacter hormaechei*	98	99.98
	sLN794248 (LN794248)	*Salmonella* Typhimurium	98	99.98
	pC45-VIM4 (LT991958)	*Enterobacter cloacae* complex	96	99.96
pS639 (*Salmonella* Thompson)	p48212_MCR (CP059413)	*Enterobacter hormaechei*	94	99.29
	pMOL665_IncHI2 (OU015720)	*Salmonella* Typhimurium	92	99.29

### Characterization of the Genetic Context Surrounding *mcr-9* Genes

**Figure 3:**
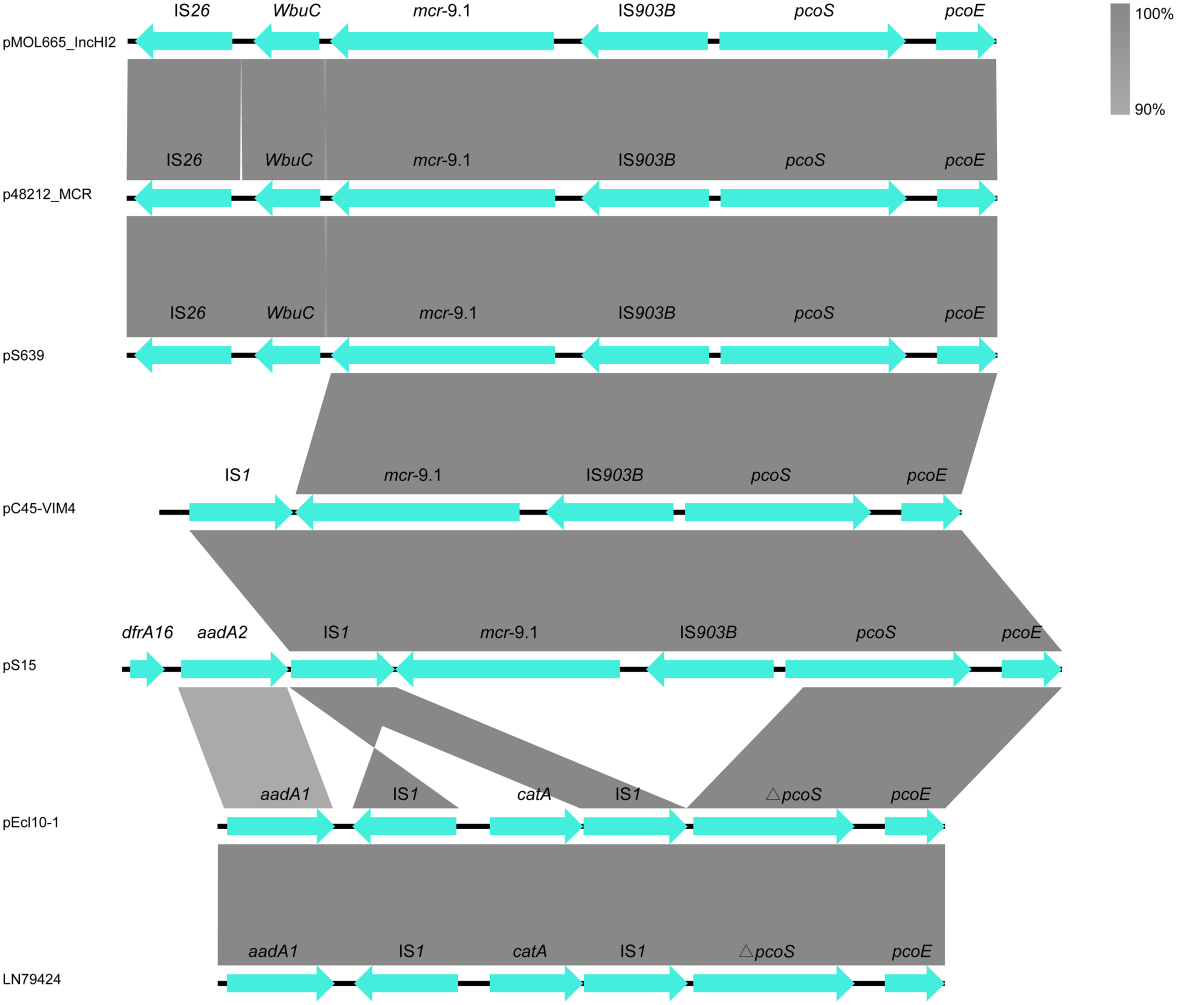
The comparison of *mcr-9* cassettes in different strains.

Our genetic analyses regarding the sequences surrounding the mcr-9 genes revealed two types (Figure 3). The mcr-9 surrounding structure of pS639 was *pcoE*-*pcoS*-IS*903B*-*mcr-9*-*WbuC*-IS*26* similar to p48212_MCR and pMOL665_IncHI2. However, in our case, the genetic context that embedded the *mcr-9* gene was *pcoE*-*pcoS*-IS*903B*-*mcr-9*-IS*1* which is present in both, the *Salmonella* plasmid pS15 and pC45-VIM4, a plasmid found in a bacterium of the Enterobacter cloacae complex. There was an insertion of IS*1* and *catA* in the position of *mcr-9* and IS*903B*.

### Transferability of *mcr-9*-Carrying Plasmids

We also tested the ability of the plasmids to be transferred to other strains and their ability to convey antibiotic resistance. We first tested the plasmid pS15 which was successfully transferred to the rifamycin-resistant *Salmonella* strain XH1984 and *E. coli* strain EC600. When determining the MICs for ampicillin, amoxicillin, piperacillin-tazobactam, and tetracycline, we found increased resistance likely conferred by the presence of the plasmid-encoded *bla*_TEM-1_ and *tet(A)* genes (Table 2). However, *mcr-9* in pS15 could not confer colistin resistance in neither *Salmonella* nor *E. coli* strains. The conjugation efficiency was calculated in pS15 plasmid conjugation assays, which was 1.9 × 10^−6^ transconjugants per donor when pS15 was transferred to XH1984 and 2.1 × 10^−8^ transferred to EC600 (Table 5).

**Table 5:** Conjugation frequency of pS15 from S15 to XH1984 and EC600.

	S15-XH1984				S15-EC600			
	1	2	3	Mean	1	2	3	Mean
Donors (D)/ml	1.1 × 10^11^	6.9 × 10^11^	5.0 × 10^10^		3.5 × 10^11^	9.0 × 10^10^	1.4 × 10^11^	
Transconjugants (TC)/ml	3.4 × 10^5^	5.7 × 10^5^	9.0 × 10^4^		8.6 × 10^3^	1.8 × 10^3^	2.5 × 10^3^	
Conjugation frequency (TC/D)	3.1 × 10^−6^	8.3 × 10^−7^	1.8 × 10^−6^	1.9 × 10^−6^	2.5 × 10^−8^	2.0 × 10^−8^	1.8 × 10^−8^	2.1 × 10^−8^

Our attempt in transferring the plasmid pS639 was unsuccessful. The reason for this is that we did not have a suitable recipient strain available which would allow the use of an antibiotic selection marker, as our strains exhibited resistance to the antibiotics encoded on the plasmid. Also, the possibility that the recipient strains were genetically not suitable to receive this specific plasmid cannot be excluded.

### Genetic Homology of *mcr-9* Carrying *Salmonella*

**Figure 4:**
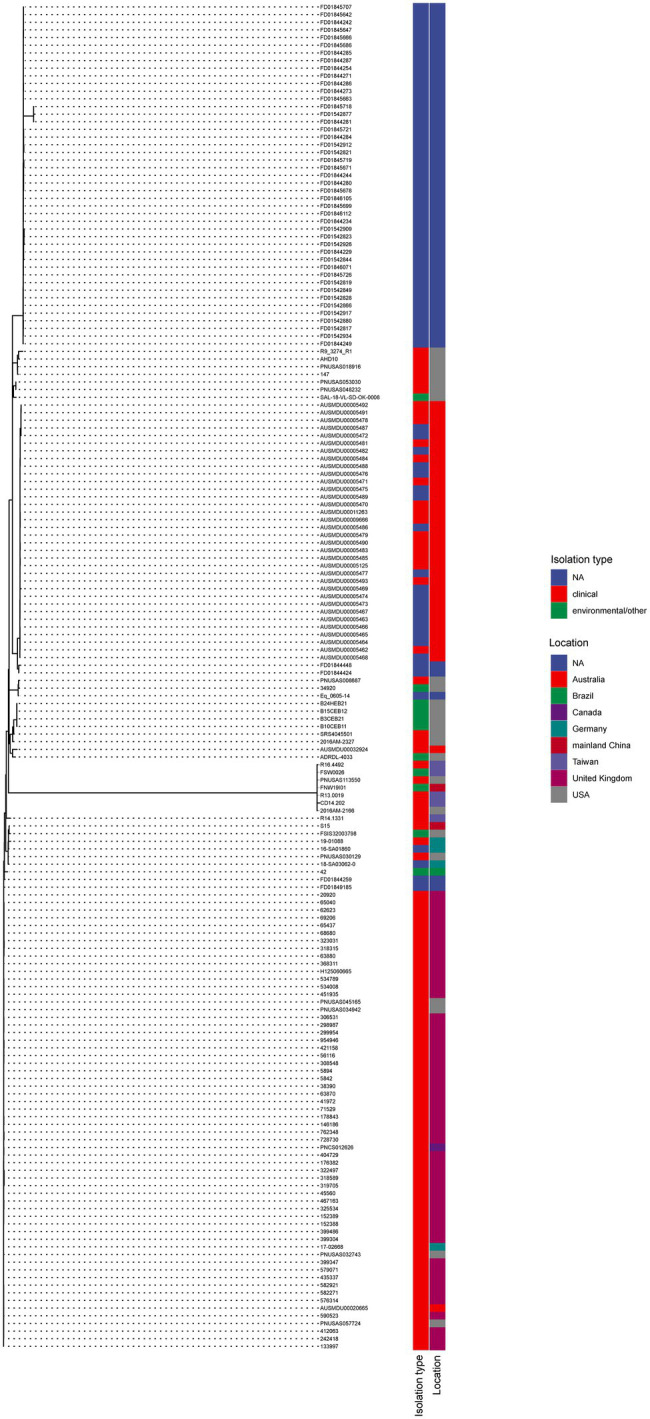
The phylogenetic tree of *mcr-9* carrying *S.* Typhimurium strains.

**Figure 5:**
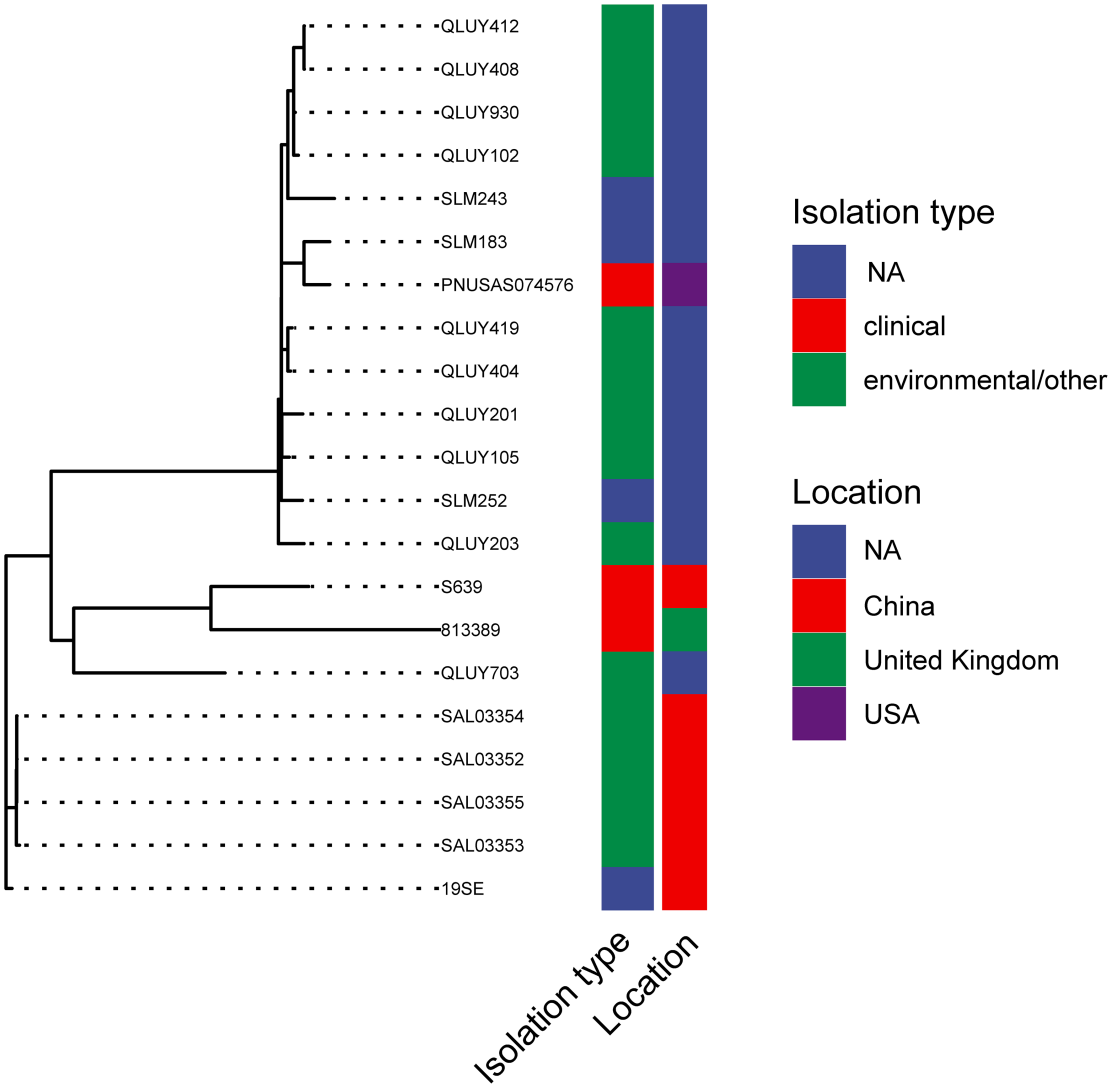
The phylogenetic tree of *mcr-9* carrying *S.* Thompson strains.

A total of 175 *S.* Typhimurium strains and 21 *S.* Thompson strains carrying *mcr-9* have been deposited in NCBI till today (February 2022). The phylogenetic trees of two serotypes were displayed in Figures 4, 5, respectively. *S.* Typhimurium strains were mostly isolated from clinical samples. Australia and the United Kingdom were the countries where the most assembled sequences were uploaded from, which does not necessarily reflect the prevalence of the strains in these countries. *S.* Typhimurium S15 was most similar to FSIS32003798 isolated from pork in the United States. As for *S.* Thompson, only three of 21 strains were clinical origin including S639, which had the closest relationship with 813,389 isolated in the United Kingdom. Interestingly, both S15 and S639 were more closely related to strains isolated outside China, despite being isolated within the country.

## Discussion

With multidrug resistance continuously increasing, colistin now belongs to the last-resort antibiotics. Plasmid-bound *mcr* alleles that mediate resistance to the antimicrobial compound are of great concern in particular if plasmid encoded due to the risk of rapid spread (Smelikova et al., 2021). Several *mcr* genes have been found, with *mcr-9* first identified in *Salmonella* Typhimurium (Carroll et al., 2019). To date, *Salmonella* strains have been reported worldwide to carry various *mcr* alleles, with mcr-1 being the most common and Typhimurium being the most prevalent serotype (Lima et al., 2019; Paveenkittiporn et al., 2021; Portes et al., 2022). As a zoonotic food-borne pathogen, *mcr*-positive *S. enterica* strains were mostly isolated from livestock, including pork and poultry, due to the fact that colistin has and continues to be used in animal husbandry (Lima et al., 2019). Therefore, it is important to monitor the spread of *mcr* alleles in *S. enterica*.

In this study, we identified two *mcr-9*-positive *Salmonella enterica* from a total of 689 clinical *Salmonella* isolates. The positive rate of *mcr-9* (0.29%) might be lower than *mcr-1* (0.87%) as we previously reported (Fan et al., 2020) although due to the low numbers (of two and six strains, respectively), statistically sound conclusions are not possible. The two *mcr-9*-positive strains belong to a different serotype, Typhimurium and Thompson. *Salmonella* Typhimurium ST34 is most commonly prevalent in causing food-borne infections in China (Wong et al., 2013). While *Salmonella* Thompson is the main serotype isolated from poultry-based products (Yang et al., 2020; Elbediwi et al., 2021b). In this study, both strains were isolated from stools of patients with diarrhea caused by *Salmonella enterica* infections, likely to have been exposed to food contaminated with the pathogen. The surprising similarity between the strains we isolated in China and those found outside the country can be explained by the rapid development of international agricultural products trade.

IncHI2 plasmids were the predominant plasmid type carrying *mcr-9* (Li et al., 2020). The two plasmids that we characterized in our study, pS15 and pS639, also belonged to the IncHI2 type, both of them having IncHI2 and IncHI2A replicons, which indicates that they are hybrid plasmids. This type of plasmid is conjugative which can result in extensive spread of the *mcr-9* gene in recipient hosts (Ai et al., 2021; Elbediwi et al., 2021a; Khodor et al., 2021; Wang et al., 2021). Testing whether the plasmids we discovered can be transmitted to other strains, we found that pS15 was indeed conjugative to both, *Salmonella* and *E. coli*. The efficiency of conjugation to *Salmonella* was about 90 times higher than that to *E. coli*, indicating pS15 was easier to spread within species. The conjugation experiments using the plasmid pS639 could not be performed due to the fact that we did not have a suitable recipient strain to our disposal as we could not select for an antibiotic encoded on the plasmid. However, pS15 and pS639 were predicted to contain similar conjugative apparatus components by oriTfinder (Li et al., 2018). Thus, it is reasonable to conclude that pS639 is likely to be conjugative since the components of the two plasmids show strong similarities.

Compared to the low level colistin resistance mediated by *mcr-1*, most of the *mcr-9*-carrying strains do not present resistance to colistin (Luo et al., 2017; Wang et al., 2021). We also observed this to be the case with the strains S15 and S639, which we described in this study. However, the inducible expression of *mcr-9* could potentially lead to an increasing of colistin MIC after exposure to low concentrations of colistin, mediated by the *qseC* and *qseB* genes (Kieffer et al., 2019). This makes *mcr-9* a gene that should not be disregarded when addressing antimicrobial resistance. Apart from this, our study identified numerous resistance genes located in the two plasmids in addition to *mcr-9*, which were responsible for the drug resistance spectrum of two strains. A total of 12 resistance genes are found in pS15 and 27 in pS639. S15 and S639 were resistant to broad spectrum penicillin and tetracycline because they both had plasmid-encoded *bla*_TEM-1_ and *tet*, which was verified by the transconjugants of pS15. In addition, pS639 encoded genes *sul*, *bla*_OXA-10_, *bla*_DHA-1_, *aacA34,* and *qnrB4*, accounting for the resistance to sulfonamides, cephalosporins, aminoglycosides, and quinolones. Since there are multiple resistance genes encoded on *mcr-9* plasmids, it is a matter of concern that co-resistance mechanism could facilitate the spread of *mcr-9*. The two types of *mcr-9* cassettes in our study, *pcoE*-*pcoS*-IS*903B*-*mcr-9*-*WbuC*-IS*26* and *pcoE*-*pcoS*-IS*903B*-*mcr-9*-IS*1*, did not include the *qseC-qseB* regulatory genes, indicating they might circulate silently. However, there might be other undetermined genes or molecules regulating *mcr-9* expression (Kananizadeh et al., 2020). Therefore, it is important to investigate the silent spread of *mcr-9* further and to monitor the dissemination of plasmids containing the colistin resistance gene.

## Data Availability Statement

The datasets presented in this study can be found in online repositories. The names of the repository/repositories and accession number(s) can be found in the article/supplementary material.

## Ethics Statement

The studies obtained ethical approval from the Ethics Committee of Hangzhou First People’s Hospital (2020103-1).

## Author Contributions

JF, YF, DZ, and XH designed the study. JF, HC, YF, LZ, JH, and YYa performed the experiments. JF, YF, LZ, JH, and HC analyzed the bioinformatics data. JF, YF, and HC wrote the manuscript. QX, DZ, SL, YYu, and XH revised the manuscript. All authors contributed to the article and approved the submitted version.

## Conflict of Interest

The authors declare that the research was conducted in the absence of any commercial or financial relationships that could be construed as a potential conflict of interest.

## Publisher’s Note

All claims expressed in this article are solely those of the authors and do not necessarily represent those of their affiliated organizations, or those of the publisher, the editors and the reviewers. Any product that may be evaluated in this article, or claim that may be made by its manufacturer, is not guaranteed or endorsed by the publisher.

## Footnotes

^1^ https://github.com/tseemann/mlst
